# A “Trial within a Cohort” platform for pediatric clinical trials on idiopathic nephrotic syndrome: scope, objectives, and design of the retrospective-prospective cohort PIN’SNP

**DOI:** 10.1007/s00467-025-06676-7

**Published:** 2025-03-03

**Authors:** Claire Bahans, Olivia Boyer, Olivier Dunand, Cyrielle Parmentier, Bruno Ranchin, Gwenaëlle Roussey, Charlotte Samaille, Stéphanie Tellier, Isabelle Vrillon, Evgenia Preka, Théo Mériguet, Astrid Dubrasquet, Lydia Ichay, Stéphanie Clavé, Julie Bernardor, Elodie Merieau, Claire Dossier, Vincent Guigonis

**Affiliations:** 1 Centre d’Investigation Clinique 1435, CHU de Limoges, Limoges, France; 2 Département de pédiatrie, CHU de Limoges, Limoges, France; 3 Néphrologie pédiatrique, Hôpital Necker-Enfants Malades, AP-HP, Paris, France; 4 Service de Néphrologie pédiatrique, CHU de La Réunion, Hôpital Félix Guyon, Saint Denis, France; 5 Néphrologie pédiatrique, Hôpital Armand Trousseau, Paris, France; 6 Néphrologie, GH Est-Hôpital Femme-Mère-Enfant, CHU de Lyon HCL, Bron, France; 7 Service de Médecine pédiatrique, Hôpital enfant-adolescent, CHU de Nantes, Nantes, France; 8 Hôpital Jeanne de Flandre, CHU de Lille, Lille, France; 9 Pédiatrie, Néphrologie, médecine interne et hypertension, Hôpital des enfants, CHU de Toulouse, Toulouse, France; 10 Service de Médecine Infantile, Unité de Néphrologie, dialyse et transplantation rénale pédiatrique -Hôpitaux de Brabois, CHU de Nancy, Vandœuvre-lès-Nancy, France; 11 Néphrologie, Hôpital des Enfants, CHU de Bordeaux, Bordeaux, France; 12 Service de Néphrologie, Hôpital Lapeyronie, CHRU Montpellier, Montpellier, France; 13 Service de néphrologie pédiatrique, Hôpital de la Timone, AP-HM, Marseille, France; 14 Service de néphrologie pédiatrique, Hôpital Archet II, CHU de Nice, Nice, France; 15 Service de néphrologie, Hôpital Clocheville, CHRU Tours, Tours, France; 16 Néphrologie pédiatrique, Hôpital Robert Debré, Paris, France

**Keywords:** Idiopathic nephrotic syndrome, Children, Cohort, Trials within cohorts, Platform

## Abstract

**Background:**

Idiopathic nephrotic syndrome (INS) in children is the most common glomerular disease and is characterized by recurrent relapses. There is no community consensus on the treatment of relapsing forms of nephrotic syndrome in children, unlike that for the initial presentation. To date, available treatments only enable relapsing patients to be maintained in remission, rather than modifying the course of the disease; therefore, more therapeutic trials are needed. The Société de Néphrologie Pédiatrique (SNP) decided to implement within its French centers a national coordinated long-term clinical research program for children treated for INS based on a Trials within Cohorts (TwiCs) model. The aim of this paper is to describe the PIN’SNP cohort and research program as well as the TwiCs design adapted to INS research in the French regulatory system.

**Methods:**

This retrospective-prospective, multicenter research program will rely on a dynamic prospective cohort of children followed for an INS, known as the PIN’SNP cohort (i) to identify cases treated within SNP centers, (ii) to describe their clinical and epidemiological characteristics, and (iii) to provide a platform to nest prospective trials, and thus facilitate inclusion of patients in these future trials.

**Conclusions:**

The PIN’SNP cohort is the first French national pediatric platform dedicated to the implementation of randomized nested trials along with longitudinal and observational studies on INS in children. The adaptation of the TwiCs design to inform all eligible patients/parents to each nested trial will facilitate methodological robustness and ethical acceptability and reinforce communication between investigators and participants.

**Trial registration:**

number NCT04207580.

**Graphical abstract:**

**Figure:**
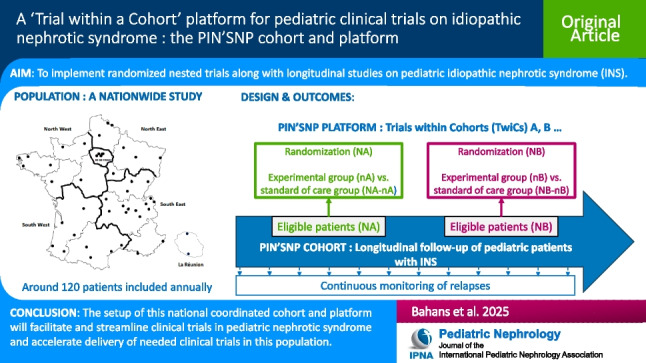
A higher-resolution version of the Graphical abstract is available as Supplementary information

**Supplementary Information:**

The online version contains supplementary material available at 10.1007/s00467-025-06676-7.

## Introduction

Idiopathic nephrotic syndrome (INS) in children is the most common pediatric glomerular disease [1]. Its incidence is estimated between 1.15 and 16.9 per 100,000 children, according to ethnicity and region [2]. There is now an international consensus on the therapeutic strategy of the first flare [2], and French guidelines have been adapted in accordance [3]. However, up to 80% of children presenting with INS will have one or more relapses [4, 5]. In contrast to the first flare, when dealing with subsequent relapses, the diversity of treatments, the limited evidence available concerning their benefit/risk ratio, and the rarity of face-to-face trials do not allow a consensus-based treatment to emerge [2]. Further therapeutic trials are thus needed, especially in the relapsing nephrotic syndrome group. Moreover, new pathophysiological hypotheses and the development of increasingly targeted treatments enable the development of strategies to modify the course of the disease rather than simply maintaining the disease in remission, as is currently the case [6].

It must be noted that there is currently, in France and worldwide, no coordinated clinical research program for a prospective evaluation of the different therapeutic strategies. Furthermore, most of the assessments focus on the short-term efficacy of treatments, more rarely on their long-term tolerance and effectiveness. For instance, the esthetic impact of treatments and patients’ quality of life are rarely considered, even though these factors may significantly affect patients’ compliance with their treatments and their experience of the disease [7]. Lastly, little data are available on the long-term outcomes of patients whose disease is no longer active in terms of impact on social, psychological, and reproductive life.

Modern management of a chronic pediatric disease can no longer ignore these aspects, and innovative research methodologies could help meet these unmet needs.

For several years, the French Société de Néphrologie Pédiatrique (SNP) has been coordinating a working group whose aim is to foster active research on INS. In addition to the prospective ongoing or achieved clinical trials coordinated by SNP members (NEPHROMYCY NCT01092962, NEPHRUTIX NCT01268033, RITUXIVIG NCT03560011, NEPHROVIR3 NCT02818738, OBIRINS NCT05786768), the group’s objective is now to develop a national coordinated long-term clinical research program for children treated for INS in SNP-affiliated centers until 18 years old [8]. This research program is based on a Trials within Cohorts (TwiCs) model and relies on a national retrospective-prospective cohort known as the PIN’SNP cohort that will enable and facilitate the implementation of multiple clinical trials within it.

Relton et al. developed the concept of TwiCs to tackle some of the difficulties associated with “classic” randomized controlled trials (RCTs): poor and slow accrual of patients, dropout rates, and limited external validity [9].

The TwiCs design uses a prospective cohort in which multiple pragmatic randomized trials may be embedded. For each trial, eligible patients are identified within the cohort and randomized between an interventional and control group. Patients of the interventional group are offered the experimental intervention, while patients of the control group still receive routine care. Data from the control group are available without any additional work since outcome measures are collected within the cohort’s usual follow-up and compared between the experimental and control groups.

In a rare chronic disease context, this design could be more efficient for conducting multiple clinical trials than implementing successive “classical” randomized controlled trials. It could, therefore, provide answers more rapidly to scientific questions specific to INS in children.

This paper aims to describe the PIN’SNP cohort and research program as well as the TwiCs design adapted to French regulatory requirements.

## Methods

### Objectives

Our objective is to create a dynamic retrospective-prospective cohort of children followed for an INS (i) to identify cases treated within SNP centers, (ii) to describe their clinical and epidemiological characteristics (presentation, treatments, complications, evolution), and (iii) to provide a basis for comparison for future trials nested within the cohort, facilitating inclusion of patients in these future trials. The first two objectives correspond to the classical objectives and definition of a cohort, while the third one can be defined as a platform for future nested trials.

### Design

This retrospective-prospective, multicenter research program concerns children presenting with INS, followed by pediatric nephrologists affiliated with the French centers of the SNP (metropolitan France, Reunion Island and Mayotte), representing 40 centers. The PIN’SNP cohort is a pediatric cohort only; patients will thus be followed until they turn 18 as long as they remain under pediatric care. The research program concerns both the PIN’SNP cohort and the platform for future nested trials. The platform will include data from patients in the PIN’SNP cohort and randomized in the control group of future nested trials.

### Eligibility criteria

Study inclusion criteria comprise the following:

1. Patient under 18 years of age
2. Diagnosis of idiopathic nephrotic syndrome (according to SNP criteria [3], i.e., including steroid-sensitive nephrotic syndrome (SSNS) and steroid-resistant nephrotic syndrome (SRNS) with no genetic origin and compatible histology at kidney biopsy) since January 1, 2020
3. Patients reviewed at least once by a pediatrician member of the SNP
4. Patients residing in France
5. Consent signed by parents and agreement to participate from the patient (if of age)
6. Affiliation with a social security scheme

### Procedures

Clinical follow-up of included patients (treatment, biological monitoring, follow-up frequency, etc.) is determined by each patient’s referring physician. Except in the case where patients are included in the treatment arm of a nested trial, inclusion in the PIN’SNP cohort only does not influence the therapeutic strategy.

The cohort consists of a retrospective and prospective, regular and systematic recording of participants’ demographic, clinical, and biological data.

Data are collected in a structured way using a pre-established form derived from:

- Consultation files as part of the usual clinical follow-up for patients with an active disease. The clinical research staff enters this information into the PIN’SNP database, validated after integration. They may ask referee investigators to validate medical data in cases where the interpretation of reports is complex. These data can be retrospectively entered into the database if the onset of the INS occurred prior to the inclusion date in the protocol
- A telephone interview for annual follow-ups for patients whose absence of active disease no longer warrants a routine consultation. This structured interview is conducted by telephone by the study’s clinical research staff
- Self- or hetero-administered quality of life questionnaires (PEDS-QL, [10]), self- or hetero-administered treatment compliance questionnaires (Morisky score, [11]), and questionnaires on some esthetic impact of treatments (Ferriman score, [12]). These annual questionnaires are centralized and entered into the database by the study’s clinical research staff

### Choice of collected data

Data collected within the cohort are presented in Table 1. They have been defined (i) to consider a broad scope of future research, whether it concerns relapses of INS or the toxicity of treatments or health outcomes, and (ii) to be limited to reduce the workload and, therefore, the cost of maintaining the cohort. A balance has been sought to address important questions related to INS treatment, while limiting the workload.

**Table 1:**
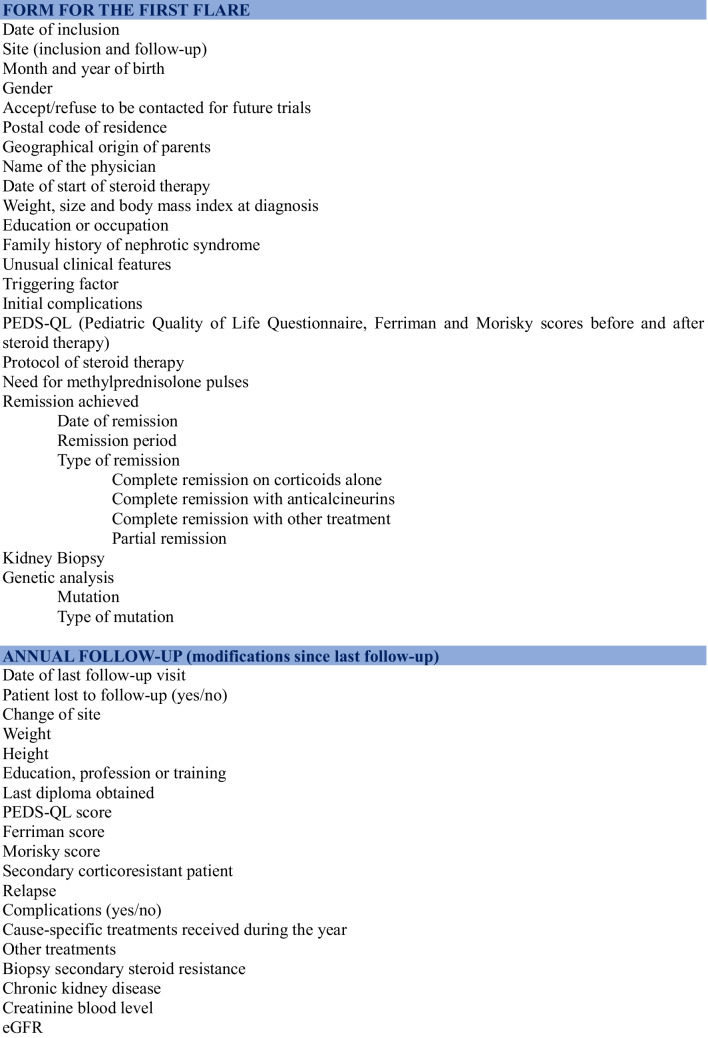

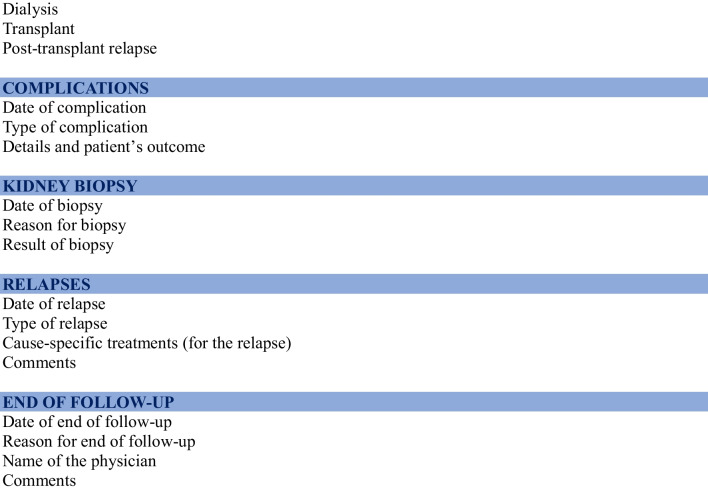
Collected data in the PIN’SNP cohort (each blue stripe is an independent form)

### Consent management

During standard-of-care hospitalization or consultation at disease onset or during a follow-up visit, investigators describe the objectives of the cohort and platform to the relevant patients and, after a period of reflection, obtain the written consent of parents and the assent of minor patients to participate in the research. To facilitate future trials nested within the PIN’SNP cohort, patients and parents are solicited (i) to be included in the cohort and (ii) to be recontacted to receive specific information on future trials for which their child could be eligible.

If patients and parents agree to be recontacted, they are informed that they will be solicited to participate in an interventional trial either in the control group or in an experimental one in the future. For each future trial, specific information and consent will be implemented.

If patients and parents do not accept to be recontacted, they are only included in the cohort and will not be solicited to participate in any nested trial.

The original TwiCs design, where patients enrolled in the control group receive no specific information, has been adapted, as required by the French Ethics Committee, to provide information, when time comes, on each nested trial to all eligible patients before randomization in the experimental and control groups.

### Data collection and management

**Fig. 1:**
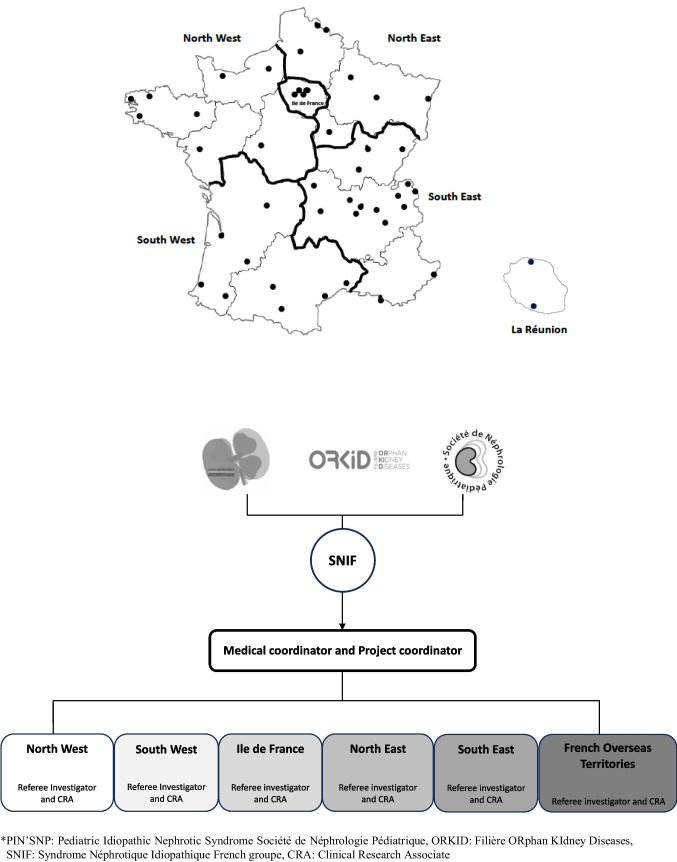
French organisation for the PIN’SNP cohort

An inter-regional organization has been implemented to facilitate inclusions, the conduct of the cohort and future nested trials (Fig. 1). France has been divided into six geographical inter-regions: Ile-de-France, North West, North East, South West, South East, and French Overseas Territories. Each inter-region is coordinated by a referee medical investigator and a clinical research associate who gathers and enters data into the database.

Study data are collected, handled, and stored in compliance with the General Data Protection Regulation (GDPR) and French law. Data are thus pseudonymized before being incorporated into the database. In this pyramidal organization, each level has access to pseudonymized data of its level or the level below (i.e., local centers have access only to the data of their center, regional coordination centers have access to data of the centers of the whole region, and only the national coordination center has access to all pseudonymized data). The Imagine Institute in Paris [13] hosts the database in a secure electronic database protected by an individual password for each investigator and clinical research associate.

Data quality is ensured by automated data entry checks using pre-defined plausibility ranges.

### Statistical aspects

It has been estimated that 200 to 300 new cases of pediatric INS are diagnosed each year in France. Considering the number of patients diagnosed in SNP-affiliated centers or secondarily referred due to subsequent relapses, the plan is to include approximately 120 new children each year in this dynamic cohort.

For the PIN’SNP cohort, usual descriptive statistics will be performed, as well as comparisons, relapse-free survival analyses, and multivariate analysis. Exploratory analyses will be conducted to study the links between the disease, the patient, and their geographical localization and to look for determinants that could predict the course of the disease or the response to treatment.

For each future nested trial within the cohort, a specific statistical analysis plan will be drafted and submitted to the French National Competent Authority and Ethics Committee for validation.

### Governance and patient involvement

The PIN’SNP platform is governed by a scientific committee composed of the PIN’SNP coordinator, the referee investigators of the French inter-regional zones, and expert members of the SNP and of the Orphan Kidney Disease network (ORKiD). The scientific committee is responsible for supervising the conduct of the PIN’SNP cohort, validating observational projects based on the cohort’s data and defining the future trials nested within the cohort. Any request for access to cohort data by independent research teams or for collaborative projects is also evaluated by the cohort’s scientific committee.

Patients are informed of the study and are willing to be involved in the planning of future research. Indeed, the French association of patients with nephrotic syndrome (AMSN) is in close contact with the PIN’SNP project’s scientific committee and actively promotes the cohort. A nested trial is currently being co-constructed to test the direct participation of patients by filling in the PIN’SNP database.

### Study progress update

The PIN’SNP cohort has been open for enrollment since March 2020, and inclusions will continue as long as funding is available. Since the setup of the cohort, 570 patients have been included in the PIN’SNP cohort and 90% of parents agree to be recontacted and informed on future nested trials.

The platform’s launch will take place as soon as funding is secured for the first nested trial. Our group is awaiting the outcome of a grant application, with results expected in February 2025.

### Ethics

A French National Research Ethics Committee approved the cohort in November 2019 (2019-A01841-56). Each future trial nested in the cohort will be submitted for authorization to the Ethics Committee and the French drug regulatory agency (Agence Nationale de Sécurité du Médicament et des produits de santé) if required.

### Funding

The PIN’SNP cohort is currently funded by the Filière des maladies rénales rares (ORKID) and the Limoges University Hospital. In the future, the economic model will have to evolve. The aim for funding the control arms of future trials (i.e., the funding of the cohort itself) is to be covered by part of the funding from successive prospective nested clinical trials.

## Discussion

The PIN’SNP cohort is a national pediatric cohort of INS patients followed by the SNP-affiliated pediatricians who collaborate efficiently together. Their expertise and the proposed organization ensure the implementation of a sustainable infrastructure to produce data for both the cohort and future nested trials. To date, 570 participants have been included in the cohort since its beginning in 2020; embedded trials will soon be implemented in parallel with ongoing inclusions. This research program offers advantages over a succession of independent trials. Thanks to the cohort, valuable ongoing and long-term information, such as the natural history of INS patients, will be available and useful for the conception of nested trials. Indeed, the statistical hypothesis for nested trials will rely on long-term data from routinely collected sources on relapses (frequency, characteristics, treatments) of pediatric INS patients in this population.

In addition, the PIN’SNP cohort will enable rapid identification and recruitment of eligible patients for nested trials and higher acceptability; it is now well known that cohorts provide more accessible and less selective recruitment [14–16]. For example, 90% of parents of PIN’SNP patients agree to be contacted and informed of future nested trials, which indicates an interest in such a design to more easily implement prospective trials.

The availability of data from the control group will save considerable time. Indeed, those data are collected as part of the PIN’SNP cohort, meaning that no additional work is required for each new nested trial.

From an administrative perspective, contracts have already been signed with the 40 investigation sites that are both open and active. The implementation of nested trials will be significantly facilitated by the contacts and contracts already in place.

The PIN’SNP cohort and platform should foster faster research in a much more efficient way than setting up successive independent trials requiring the opening and closing of sites, the implementation of a new data collection system, and the lengthy recruitment process for each trial.

There is an increasing number and maturity in RCTs conducted within cohorts in a wide range of conditions, such as oncology and mental and behavioral disorders [17]. INS is a condition well-suited for this study design as a chronic illness, well-phenotyped, prone to relapse, and with well-defined subgroups of patients according to international recommendations. Considering all these criteria, the same patient may be allowed to participate in several successive trials throughout his or her treatment.

The International Pediatric Nephrology Association (IPNA) recently published a list of future research within its recommendations for the Diagnosis and Management of Children with Steroid Sensitive Nephrotic Syndrome [2]. Among these recommendations, research that could be implemented using the PIN’SNP platform is presented in Table 2. A wide range of topics can be investigated regarding relapse prevention, sparing treatments, vaccination, or quality of life. The platform will, therefore, enable research to be developed in all areas of INS.

**Table 2:** IPNA Future Research recommendations with evaluation of implementation in the PIN’SNP TwiCs platform

Topic	Subtopic	Research question	Feasibility in PIN’SNP
First episode of nephrotic syndrome (NS)	Treatment with prednisolone (PDN)	Compare the effectiveness of treatment with oral PDN for 8 (4 + 4) weeks or shorter duration vs. 12 (6 + 6) weeks (daily/alternate daily PDN) in terms of outcomes such as time to first relapse, frequently relapsing NS (FRNS) and steroid-dependent NS (SDNS)	Yes if patients are included at the time of the first episode of NS, otherwise no.
		Compare the effectiveness of initial treatment with 30 mg/m^2^(1 mg/kg) for 4 weeks and alt day for 4 wks with 60 mg/m^2^ (2 mg/kg) for 4 weeks and alt day for 4 wks	
		Determine if initial PDN duration >12 weeks affects future disease course in very young children	
	PDN dosing	Evaluate the dosing of PDN by weight or body surface area (BSA) for outcomes of effectiveness such as inducing remission, time to first relapse, FRNS, SDNS, and steroid toxicity	Yes if patients are included at the time of the first episode of NS, otherwise no
	Steroid-sparing agent	Assess combination therapy of PDN with a steroid-sparing agent at disease onset to determine effectiveness of reducing time to remission, increasing time to first relapse or development of FRNS or SDNS	Yes if patients are included at the time of the first episode of NS, otherwise no
	Pharmacology, pharmacokinetics, pharmacogenomics	Determine the mode of action of glucocorticoids and other immunosuppressive medications in steroid-sensitive NS (SSNS)	Yes if patients are included at the time of the first episode of NS, otherwise no
		Examine the pharmacokinetics of prednisone by age	
		Determine the role of pharmacogenomics in guiding selection of dose and duration of prednisone, and second-line immunosuppressive agents	
	Risk stratification	Identify biomarkers or genetic risk haplotypes to stratify disease subgroups and to assist in selection of appropriate therapeutic agents	Yes if patients are included at the time of the first episode of NS, otherwise no
Relapses	Treatment with PDN	Determine the minimum dose and duration of PDN for treatment of steroid-sensitive relapses in order to regain and maintain remission, reduce PDN exposure, toxicity, and improve quality of life	Yes
	PDN dosing	Evaluate the effectiveness of dosing of prednisone by weight or BSA in inducing remission and reducing steroid toxicity	Yes
	Low dose daily versus low-dose alternate day PDN dosing	Compare the efficacy and safety of low-dose daily versus low-dose alternate day PDN dosing as long-term maintenance treatment to prevent relapses	Yes
	Prevention of relapses	Optimize treatment protocols for SSNS after relapse according to clinical phenotypes addressing important demographic variables such as age, sex, and ethnicity	Yes
		Determine if administering PDN treatment at the start of an infection is effective to maintain remission and prevent relapse	
	Prevention of upper respiratory tract infection (URTI) associated relapses	Determine the effectiveness of short term escalation of immunosuppression for prevention of URTI associated relapses in infrequently relapsing NS (IRNS), FRNS, SDNS	Yes
		Determination of the risk of URTI-associated relapse for large cohorts of children to understand differences according to ethnicity, geography and disease course	
	Adrenal function	Evaluate prevalence and incidence of adrenal insufficiency in children with SSNS at different points in their disease history, in terms of both symptoms of adrenal insufficiency and its impact on risk of relapses	Yes
	General impact of relapses	Measure the pattern of relapses in different populations to better understand the incidence of complex relapses and the impact of relapses on quality of life and health economics	Yes
Frequently relapsing nephrotic syndrome (FRNS)/steroid-dependent nephrotic syndrome (SDNS)	Treatment with steroid-sparing agents	Compare the efficacy of different immunosuppressive therapies in maintaining sustained remission and reducing the frequency of relapses in order to determine how and when the different immunosuppressive therapies should be used	Yes
		Perform a RCT assessing antiCD20 agents, such as obinutuzumab, belimumab, daratumumab in comparison to RTX or as adjunctive therapy to other steroid-sparing agents	
	Duration of treatment	Determine the optimal duration of therapies of levamisole, mycophenolate mofetil, calcineurin-inhibitors	Yes
	Levamisole—side effect	Assess the risk of Anti-Neutrophilic Cytoplasmic Autoantibody (ANCA) positive vasculitis	Yes
	Mycophenolate mofetil (MMF)—drug monitoring	Determine the utility and benefits of drug monitoring	Yes
	Rituximab—safety, dosing, monitoring	Determine the safety of therapy with rituximab (RTX), specifically the risk of transient or sustained hypogammaglobulinemia, and other serious adverse effects	No
		Determine the optimal RTX individual dose in children	Yes
		Examine the efficacy of sequential administration of RTX in maintaining remission and safety in order to determine the optimal number and timing of RTX retreatment	Yes
		Examine the immune phenotype of B-lymphocytes following rituximab-induced remission and during relapse	Yes
		Evaluate the importance of monitoring for RTX plasma levels and anti-chimeric RTX antibodies	Yes
Drug toxicity		Devise validated objective scores to measure acute and chronic corticosteroid toxicity	No
		Compare toxicity from corticosteroids and non-steroid immunosuppression to help guide changes in maintenance treatment	
Genetics	Familial steroid-sensitive nephrotic syndrome (SSNS)	Examine the genetic basis of SSNS, focusing on families with steroid sensitive disease	Yes
Adjunctive measures	Vaccination	Determine the efficacy and safety of live attenuated vaccines in children on maintenance immunosuppressive therapy	Yes
	Edema	Determine the efficacy of albumin and/or diuretics in the management of severe edema	Yes
Health outcomes	Quality of life	Assess the quality of life in all clinical trials as a patient-centered endpoint	Yes
	Long-term safety	Assess the cumulative risk of late side effects from NS immunosuppression therapy	Yes, but only for side effects that are usually and systematically reported in medical file reports
	Adult outcome	Assess the impact of childhood onset NS in adulthood	Yes

Despite all its advantages, the PIN’SNP platform design will not systematically replace all future classical randomized controlled trials. The future implemented trials in the cohort will be pragmatic trials centered on effectiveness and rely on data collected from routine care for the control group. They will, therefore, be studies complementary to “conventional” randomized controlled trials, where experimental conditions can be fully controlled. The PIN’SNP platform is not built to run explanatory trials centered on efficacy with double-blinding and placebo. In addition, given the network on which this cohort is built (mostly third level centers of French pediatric nephrology units), relapsing patients will be over-represented among all INS patients included, as patients with no relapse are rarely referred to a reference center, if they live far from it. Therefore, the PIN’SNP platform is not designed to run a population-based cohort study.

However, this complementary approach will be based on a robust methodology, and the results will be reported in compliance with the CONSORT routine, developed explicitly for reporting randomized controlled trials using cohorts and routinely collected data [14].

PIN’SNP is the first and unique French cohort to follow pediatric patients presenting with INS all over France and overseas French territories. NEPHROVIR-1 was a fixed population-based cohort of INS children aged 6 months to 16 years old and residing in Ile-de-France between December 2007 and May 2010 [5]. The NEPHROVIR cohort and follow-up provided exhaustive but geographically limited data. In contrast, the PIN’SNP cohort covers the whole French territory, thus providing more significant though non-exhaustive data on French pediatric patients. At a European level, the PodoNet Registry is focused on patients with congenital and steroid-resistant nephrotic syndrome and collects clinical and genetic information to establish genotype–phenotype correlations in hereditary forms of the disease; its scope is therefore different [18]. The European Rare Kidney Disease Registry (ERKReg) is a recent web-based registry for all patients with rare kidney diseases, which comprises more than 300 inherited, congenital, or acquired disorders, including INS [19]. Primary and longitudinal data are collected to provide disease and treatment information from all patients with rare kidney diseases followed at expert centers. The ERK Registry is a valuable tool but does not have the same scope as the PIN’SNP cohort. The ERK registry also plans to serve as a platform for sub-registries to collect more detailed information on rare kidney diseases or disease groups. In the future, it will probably be interesting for our group to connect with the ERK registry to implement European clinical trials.

In Canada, the regional INSIGHT cohort is an observational longitudinal study on childhood nephrotic syndrome focused on clinical, genetic, and environmental factors influencing susceptibility to nephrotic syndrome and progression to chronic kidney disease among a multi-ethnic group of children [20]. The INSIGHT cohort is limited to Ontario and has not been designed as a platform for prospective nested trials. The topics studied in this Canadian cohort will provide valuable epidemiological information that will complement our team’s trials.

In the UK, the National Unified Renal Translational Research Enterprise: Idiopathic Nephrotic Syndrome study (NURTuRE-INS [21]) is a prospective cohort study of children and adults with biosamples and detailed clinical data for 739 INS patients from across the UK. The NURTuRE-INS cohort aims to pioneer innovative approaches for stratifying INS patients, deepening insights into the disease, and advancing research into its underlying mechanisms. The work conducted within this cohort complements that of our team and will undoubtedly contribute to the design of future nested trials within the PIN’SNP cohort.

While the PIN’SNP cohort is designed with a nationwide scope, it will inevitably face challenges in addressing rare or extremely rare conditions (e.g., INS in very young infants or anti-CD20-resistant NS). An international network could be envisioned, built upon the collaboration of several—or potentially all—of the previously mentioned cohorts. But before including a first patient in a prospective study within such a consortium of cohorts, many issues will have to be addressed such as international funding, patient consent management based on national regulations (included international use of e-consent), and coordination of reglementary approvals across different countries. Given the challenges associated with such tasks, we plan to start at the national level before considering moving to a European and/or international level.

The PIN’SNP platform features several methodological modifications compared with a conventional TwiCs design. In classical TwiCs studies, information is provided only to the patients randomized in the experimental arm. As a result, only patients in the experimental group can refuse the intervention. This refusal can lead to non-compliance within the experimental arm, constituting a methodological challenge that requires careful sample size planning. Additionally, it can introduce bias in estimating the average causal effect of the received treatment [16]. In the PIN’SNP platform, as information will be given to both control and interventional arms, non-compliance will likely be randomly distributed over study arms. It will not be necessary to manage the anticipated non-compliance rate in the intervention group and how it affects the choice and definition of the analysis methods and the sample size calculation or adaptation [16].

We are well aware of the inherent limitations of such a platform. Indeed, to date, the data quality of the PIN’SNP cohort only relies on automated data entry checks using pre-defined plausibility ranges, which prevents the saving of implausible data. We are currently working on more refined automatic consistency checks and queries regularly, to ensure reliable and robust data.

The challenge for PIN’SNP is the long-term sustainability of the cohort and platform. Financial and human resources necessary to collect, manage, and analyze data and to conceive and implement future nested trials are substantial, especially for academic research teams. Calls for projects are most often made for short-term funding, whereas this type of research is based on the long term. Research funding systems should be adapted to such new and innovative research methods. Another challenge will be to keep patients motivated to continue being cohort members. Communication based on feedback and newsletters should be implemented, as well as events specifically dedicated to them. In addition, as a complementary tool to the information provided by investigators, our team is currently deploying electronic information and consent to provide information to patients between consultations. The implementation of e-consent is new and, therefore, a significant challenge for all French stakeholders: sponsors have to find reliable and secure devices, investigators and patients need time to get used to electronic tools while ethics committees and the French data protection agency must check the acceptability and security of the process and devices.

In conclusion, the PIN’SNP cohort is the first French national pediatric platform dedicated to implementing randomized nested trials and longitudinal and observational studies on INS in children. Adapting the TwiCs design to inform all eligible patients/parents about each nested trial will facilitate methodological robustness and ethical acceptability and reinforce communication between investigators and participants.

## Supplementary Information

Below is the link to the electronic supplementary material.

Graphical abstract (PPTX 134 KB)
