# Supplementary material for: A “Trial within a Cohort” platform for pediatric clinical trials on idiopathic nephrotic syndrome: scope, objectives, and design of the retrospective-prospective cohort PIN’SNP
Source: Pediatr Nephrol. 2025 Mar 3;40(7):2225–38. doi: 10.1007/s00467-025-06676-7 (PMC12117012; doi:10.1007/s00467-025-06676-7)
Supplement: Supplementary file 1 — Graphical abstract (PPTX 134 KB) [file 467_2025_6676_MOESM1_ESM.pptx]

## Slide 1
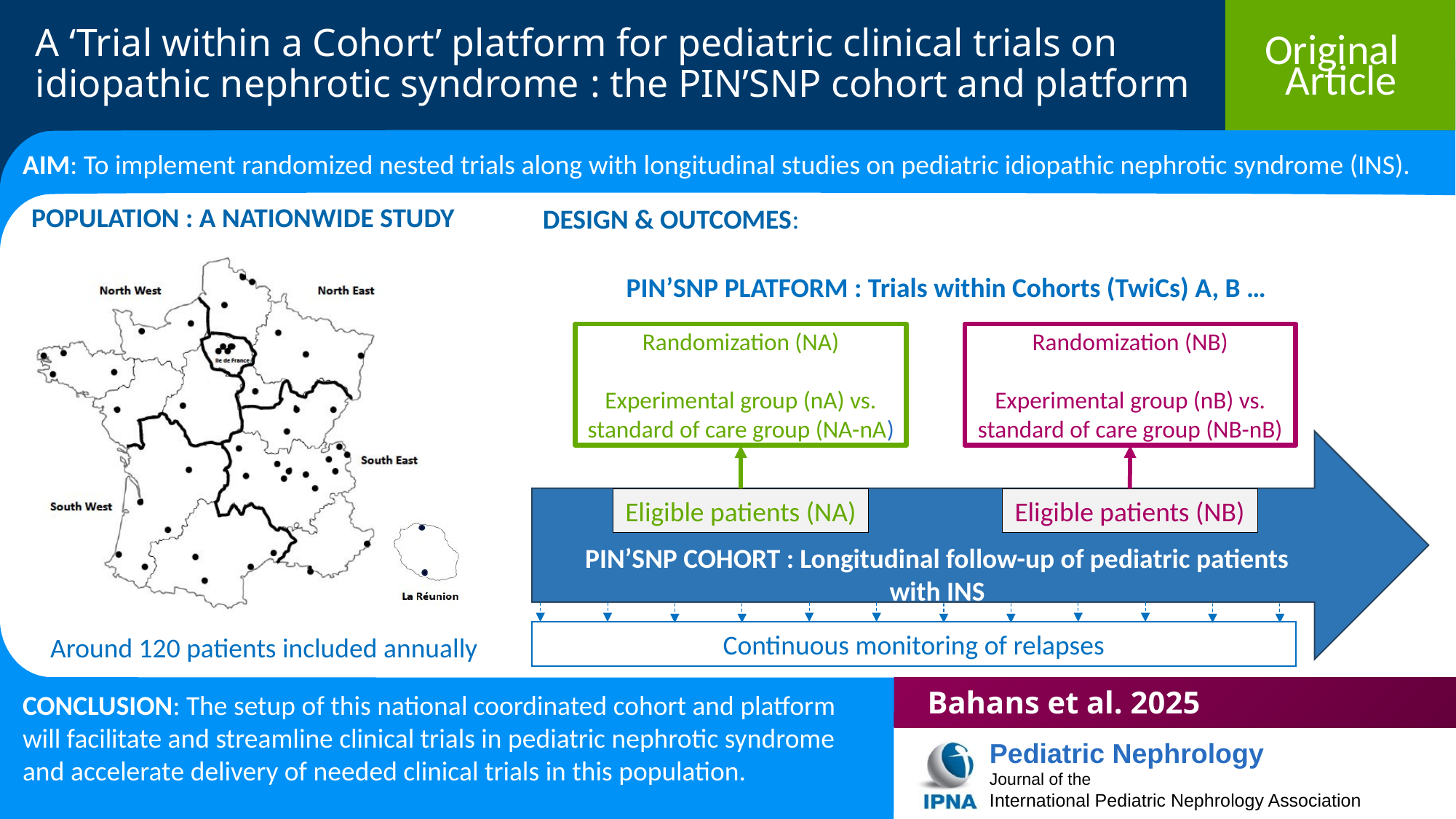

A ‘Trial within a Cohort’ platform for pediatric clinical trials on idiopathic nephrotic syndrome : the PIN’SNP cohort and platform
AIM: To implement randomized nested trials along with longitudinal studies on pediatric idiopathic nephrotic syndrome (INS).
POPULATION : A NATIONWIDE STUDY
DESIGN & OUTCOMES:
PIN’SNP PLATFORM : Trials within Cohorts (TwiCs) A, B …
Randomization (NA)
Experimental group (nA) vs. standard of care group (NA-nA)
Randomization (NB)
Experimental group (nB) vs. standard of care group (NB-nB)
Eligible patients (NA)
Eligible patients (NB)
PIN’SNP COHORT : Longitudinal follow-up of pediatric patients with INS
Continuous monitoring of relapses
Around 120 patients included annually
Bahans et al. 2025
CONCLUSION: The setup of this national coordinated cohort and platform will facilitate and streamline clinical trials in pediatric nephrotic syndrome and accelerate delivery of needed clinical trials in this population.
